# *Punica granatum *(Pomegranate) juice provides an HIV-1 entry inhibitor and candidate topical microbicide

**DOI:** 10.1186/1471-2334-4-41

**Published:** 2004-10-14

**Authors:** A Robert Neurath, Nathan Strick, Yun-Yao Li, Asim K Debnath

**Affiliations:** 1 Biochemical Virology Laboratory, Lindsley F. Kimball Research Institute, New York Blood Center, New York, USA; 2 Laboratory of Molecular Modeling & Drug Design, Lindsley F. Kimball Research Institute, New York Blood Center, New York, USA

## Abstract

**Background:**

For ≈ 24 years the AIDS pandemic has claimed ≈ 30 million lives, causing ≈ 14,000 new HIV-1 infections daily worldwide in 2003. About 80% of infections occur by heterosexual transmission. In the absence of vaccines, topical microbicides, expected to block virus transmission, offer hope for controlling the pandemic. Antiretroviral chemotherapeutics have decreased AIDS mortality in industrialized countries, but only minimally in developing countries. To prevent an analogous dichotomy, microbicides should be: acceptable; accessible; affordable; and accelerative in transition from development to marketing. Already marketed pharmaceutical excipients or foods, with established safety records and adequate anti-HIV-1 activity, may provide this option.

**Methods:**

Fruit juices were screened for inhibitory activity against HIV-1 IIIB using CD4 and CXCR4 as cell receptors. The best juice was tested for inhibition of: (1) infection by HIV-1 BaL, utilizing CCR5 as the cellular coreceptor; and (2) binding of gp120 IIIB and gp120 BaL, respectively, to CXCR4 and CCR5. To remove most colored juice components, the adsorption of the effective ingredient(s) to dispersible excipients and other foods was investigated. A selected complex was assayed for inhibition of infection by primary HIV-1 isolates.

**Results:**

HIV-1 entry inhibitors from pomegranate juice adsorb onto corn starch. The resulting complex blocks virus binding to CD4 and CXCR4/CCR5 and inhibits infection by primary virus clades A to G and group O.

**Conclusion:**

These results suggest the possibility of producing an anti-HIV-1 microbicide from inexpensive, widely available sources, whose safety has been established throughout centuries, provided that its quality is adequately standardized and monitored.

## Background

The global AIDS epidemic has proceeded relentlessly for ≈ 24 years with no promising prophylactic intervention in sight. In 2003 there were 5 million new HIV infections, and 3 million AIDS deaths [1]. To date the number of individuals living with HIV-1 infection/AIDS has reached 40 million, and ≈ 30 million people have already died from AIDS since the beginning of the pandemic [1,2]. Most new infections have been acquired by the mucosal route, heterosexual transmission playing the major (≈ 80%) role. Although the incidence of transmission per unprotected coital act is estimated to be low (0.0001 – 0.004), but strikingly increased when acutely infected individuals are involved [3,4], the cumulative effect is overwhelming.

Anti-HIV-1 vaccines applicable to global immunization programs are not expected to become available for many years. Thus, other prevention strategies are urgently needed. This includes educational efforts and application of mechanical and/or chemical barrier methods. The latter correspond to microbicides, i.e. topical formulations designed to block HIV-1 infection (and possibly transmission of other sexually transmitted diseases) when applied vaginally (and possibly rectally) before intercourse [3,5-7]. Conceptually, it is preferred that the active ingredient(s) of microbicide formulations (1) block virus entry into susceptible cells by preventing HIV-1 binding to the cellular receptor CD4, the coreceptors CXCR4/CCR5 and to receptors on dendritic/migratory cells (capturing and transmitting virus to cells which are directly involved in virus replication), respectively [3,8-11], and/or (2) are virucidal. The formulations must not adversely affect the target tissues, and should not cause them to become more susceptible to infection after microbicide removal [12,13].

Treatment with anti-retroviral drugs has decreased mortality from AIDS in industrialized countries but has had a minimal effect so far in developing countries [14]. To avoid a similar dichotomy with respect to microbicides, they should be designed and selected to become affordable and widely accessible, while shortening the time between research and development and their marketing and distribution as much as possible. This would be facilitated if mass manufactured products with established safety records were to be found to have anti-HIV-1 activity. Qualifying candidates to be considered for microbicide development may possibly be discovered by screening pharmaceutical excipients (= "inactive" ingredients of pharmaceutical dosage forms) and foods, respectively, for anti-viral properties. This approach has already led to the discovery of cellulose acetate 1,2-benzenedicarboxylate (used for coating of enteric tablets and capsules) as a promising candidate microbicide [15-19]. Here we report the outcome of screening fruit juices neutralized to pH ≈ 7 to discount nonspecific effects caused by acidity.

## Methods

### Reagents

Pomegranate juices (PJ) were purchased in local New York City stores; their origin is given in parentheses: PJ1 (Madeira Enterprises Inc., Madeira, CA); PJ2 was prepared from fresh ripe pomegranates in our laboratory; PJ3 (Sadaf^®^; Sadaf^® ^Foods, Los Angeles, CA; additional ingredients: fructose, citric acid); PJ4 (Cortas Canning & Refrigeration Co. S.A.L., Beirut, Lebanon); PJ5 (Kradjian, Import & Wholesale Distribution, Glendale, CA. Product of Iran); PJ6 (R.W. Knudsen ; Just Pomegranate; Knudsen & Sons, Inc., Chico, CA); PJ7 (Aromaproduct Ltd., Product of Georgia; distributed by Tamani, Inc., New York, NY). Starches used were: PURE-DENT^® ^B815 Corn Starch NF, PURE-DENT^® ^B816 Corn Starch USP, Spress^® ^B825 Pregelatinized corn starch NF, Spress^® ^B820 Pregelatinized corn starch NF, INSTANT PURE-COTE™ B792 Food starch-modified, INSCOSITY™ B656 Food starch-modified (Grain Processing Corporation, Muscatine, IN); PURITY^® ^21 corn starch NF and PURITY^® ^826 corn starch NF (National Starch and Chemical Company, Bridgewater, NJ); Remyline AX-DR Waxy rice starch and Remy DR native rice starch, medium grind (A&B Ingredients, Fairfield, NJ); ARGO^® ^corn starch (Best Foods Division, CPC International Inc., Engelwood Cliffs, NJ); STALEY^® ^pure food powder starch (Tate & Lyle, Decatur, IL); STARCH 1500 pregelatinized starch NF (Colorcon, West Point, PA). The following polymers were used: polyethylene glycols (PEG) 1000 NF, 1500 NF and 8000 NF; and hydroxypropyl methylcellulose, 50 cps, USP (Spectrum, New Brunswick, NJ); Carbopol 974P-NF (B. F. Goodrich Co., Cleveland, OH); Carbophil, Noveon AA1 (Noveon, Inc., Cleveland OH); and Pharmaburst B2 (SPI Pharma, New Castle, DE). Fattibase was from Paddock Laboratories, Inc., Minneapolis, MN.

Recombinant proteins employed were: HIV-1 IIIB gp120, biotinyl-HIV-1 IIIB gp120, CD4, and biotinyl-CD4 (ImmunoDiagnostics, Inc., Woburn, MA); HIV-1 IIIB BaL gp120 and FLSC (a full length single chain protein consisting of BaL gp120 linked with the D1D2 domains of CD4 by a 20 amino acid linker) (produced in transfected 293T cells [20]). Phycoerythrin (PE)-labeled streptavidin was from R & D Systems, Minneapolis, MN. Biotinylated *Galanthus nivalis *lectin was from EY Laboratories, Inc. San Mateo, CA. Rabbit antibodies to synthetic peptides from gp120 (residue numbering as in reference [21]) were prepared as described [21]. Monoclonal antibodies (mAb) 588D, specific for the CD4 binding site, and 9284, specific for the gp120 V3 loop, were from Dr. S. Zolla-Pazner and NEN Research Products, Du Pont, Boston, MA, respectively. A "generic" version of the nonnucleoside HIV-1 reverse transcriptase inhibitor TMC-120 [22] was synthesized by Albany Molecular Research, Inc., Albany, NY, and used in control experiments at a final 5 μM concentration. Pelletted, 1000-fold concentrates of HIV-1 IIIB (6.8 × 10^10 ^virus particles/ml) and BaL (2.47 × 10^10 ^virus particles/ml) were from Advanced Biotechnologies, Inc., Columbia, MD. Primary HIV-1 isolates, MT-2 cells, HeLa-CD4-LTR-β-gal and U373-MAGI-CCR5E cells (both contributed by Dr. Michael Emerman) and Cf2Th/synCCR5 cells (contributed by Dr. Tajib Mirzabekov and Dr. Joseph Sodroski) were obtained from the AIDS Research and Reference Reagent Program operated by McKesson BioServices Corporation, Rockville, MD. CEMx174 5.25M7 cells, transduced with an HIV-1 long terminal repeat (LTR)-green fluorescent protein and luciferase reporter construct, expressing CD4 and CXCR4 and CCR5 coreceptors [23], were obtained from Dr. Cecilia Cheng-Mayer. The cells were maintained in RPMI-1640 medium supplemented with 10% fetal bovine serum (FBS), 1 μg/ml puromycin and 200 μg/ml G418. These cells are suitable for titration of both X4 and R5 HIV-1 isolates and for determining the effectiveness of anti-HIV-1 drugs with reliable reproducibility. This is impossible to accomplish by using peripheral blood mononuclear cells (PBMCs) because of their variations in susceptibility to HIV-1 infection among cells derived from distinct individuals [24-26]. PBMCs were isolated from HIV-1 negative donors as described [27].

### Formulations

In attempts to separate gp120-CD4 binding inhibitory activity from most other ingredients of PJ, 200 mg of distinct starch preparations were added per ml of PJ1. After mixing for 1 h at 20°C, excess juice was decanted, and the pellets resuspended in 1 ml of distilled water. Based on results of enzyme linked immunosorbent assays (ELISA), PURITY^® ^21 corn starch, NF grade (S21) was selected for further studies, and the corresponding PJ complex was designated as PJ-S21. PJ-S21 was freeze dried and used to prepare the following formulations: PEG suppositories (50% PJ-S21, 45% PEG 1000, 5% PEG 1500); Fattibase suppositories (50% PJ-S21, 50% Fattibase); and mucoadhesive instantly dispersible tablets (50% PJ-S21, 20% HPMC, 20% Pharmaburst, 7.5% Carbopol 974P and 2.5% Carbophil).

### Enzyme linked immunosorbent assays (ELISA)

Inhibition of infection by HIV-1 IIIB and BaL, respectively, was determined relying on a β-galactosidase readout system [18]. The enzyme was quantitated with a Galacto-Light Plus System chemiluminescence reporter assay (Applied Biosystems, Foster City, CA) using a Microlight ML 2250 luminometer (Dynatech Laboratories, Inc., Chantilly, VA). To measure virucidal activity, virus was separated from excess inactivating agent by centrifugation and/or precipitation with PEG 8000 [18,19]. Serial dilutions of the treated virus were assayed for infectivity as described above. Dose response curves (i.e. luminescence vs. dilution) for treated and control viruses were obtained, and the percentages of virus inactivation were calculated [19]. To determine inhibition of infection by primary HIV-1 strains, CEMx174 5.25 M7 cells were incubated with 100 × TCID_50 _of primary HIV-1 strains in the absence or presence of PJ-S21 at graded concentrations for 3 days at 37°C. The experiments were done in triplicate. Infection was quantitated by measuring luciferase activity [23] using a kit from Promega (Madison, WI) in an Ultra 384 luminometer (Tecan, Research Triangle Park, NC).

CD4-HIV-1 gp120 binding and its inhibition were measured by ELISA. Wells of 96-well polystyrene plates (Immulon II, Dynatech Laboratories, Inc., Chantilly, VA) were coated with 100 ng/well of either gp120 IIIB or gp120 BaL, and post-coated as described [16]. Dilutions of PJs and of PJ-S21, respectively, in 0.14 M NaCl, 0.01 M Tris, 0.02% sodium merthiolate, pH 7.0 (TS) containing 100 μg/ml bovine serum albumin (BSA) were added to the wells for 1 h at 37°C. The wells were washed 5 × with TS. Biotinyl-CD4 (1 μg/ml) in TS-1% gelatin was added to the wells for 5 h at 37°C. After washing 1 × with TS-0.1% Tween 20 and 5 × with TS, horseradish peroxidase (HRP)-streptavidin (0.625 μg/ml; Amersham, Arlington Heights, IL) in TS-2% gelatin-0.05% Tween 20 was added. After 30 min at 37°C, the wells were washed 4 × with TS-0.1% Tween 20 and 2 × with TS. Bound HRP was detected using a kit from Kirkegaard and Perry Laboratories Inc. (Gaithersburg, MD) and the absorbance (A) read at 450 nm. A in the absence of inhibitors was 1.0 to 1.5, and 0 to 0.005 in the absence of biotinyl-CD4. In an alternative assay, CD4 (500 ng/ml) was mixed with biotinyl-gp120 (1 μg/ml) in the presence or absence of inhibitors for 30 min at 20°C. Serial dilutions of the mixtures were added to wells coated with the anti-CD4 mAb OKT 4 (Ortho-Clinical Diagnostics, Rochester, NY) and captured biotinyl-gp120 was detected with HRP-streptavidin as described above. To measure binding to gp120 of antibodies to gp120 peptides, the respective rabbit antisera were diluted 50-fold in a mixture of FBS and goat serum (9:1) containing 0.1% Tween 20 (pH 8.0) and added to gp120 wells. Bound IgG was detected with HRP labeled anti-rabbit IgG (Sigma, St. Louis, MO; 1 μg/ml in TS-10% goat serum-0.1% Tween 20). A cell-based ELISA was used to measure the blocking of CCR5 binding sites on HIV-1 BaL gp120 by PJ and PJ-S21, respectively [20]. Briefly, FLSC (125 ng/ml) in the absence or presence of graded amounts of inhibitors was added to Cf2Th/synCCR5 cells fixed with 5% formaldehyde in wells of 96-well plates. After 1 h at 37°C, bound FLSC was detected with mAb M-T441 (125 ng/ml; Ancell, Bayport, MN) specific for the CD4 D2 domain, followed sequentially by biotinylated anti-mouse IgG and HRP-streptavidin.

## Results

### Anti-HIV-1 activity of pomegranate juice

**Figure 1:**
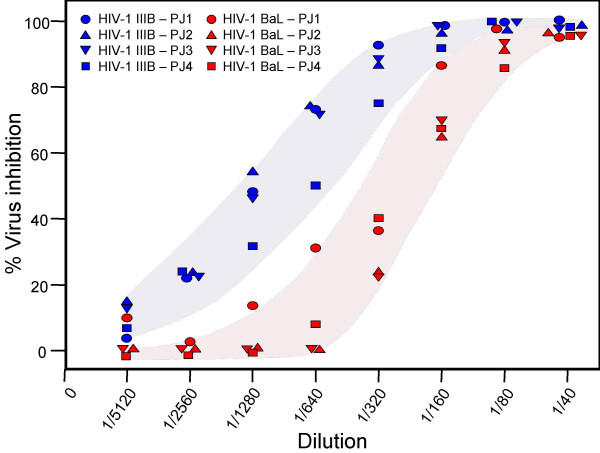
**Inhibition of HIV-1 infection of HeLa-CD4-LTR-β-gal and U373-MAGI-CCR5E cells, respectively, by pomegranate juice (PJ). **Blue shaded area = HIV-1 IIIB; red shaded area = HIV-1 BaL. Four distinct PJs (PJ1 to PJ4) were tested. Infection was monitored by measuring β-galactosidase.

Serial twofold dilutions of juices [apple, black cherry, blueberry, coconut milk, cranberry, elderberry, grape (red), grapefruit, honey, lemon, lime, pineapple, pomegranate and red beet (10% reconstituted dry powder)] were assayed for inhibition of infection by HIV-1 IIIB of cells expressing the CD4 and CXCR4 receptors and coreceptors. Most juices (4-fold diluted) had no inhibitory activity, except blueberry, cranberry, grape and lime juice, respectively [endpoints for 50% inhibition of infection (ED_50_) between 1/16 and 1/64]. Consistently, PJs from distinct geographical areas had the highest inhibitory activity (Fig. 1; blue shaded area). Since HIV-1 viruses utilizing CCR5 as coreceptor (=R5 viruses) are predominantly transmitted sexually [3,28], it was important to test whether PJ can inhibit not only infection by HIV-1 IIIB, a virus utilizing CXCR4 as coreceptor (=X4 virus), but also infection by an R5 virus, HIV-1 BaL. Results in Fig. 1 (red shaded area) show that infection by the latter virus is also inhibited, albeit less effectively than that by HIV-1 IIIB.

**Figure 2:**
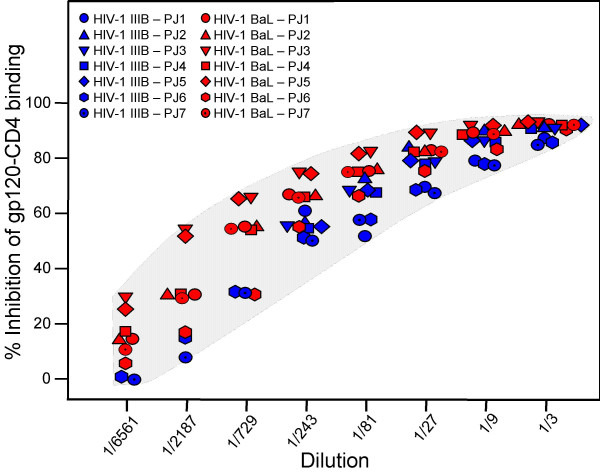
**Inhibition of CD4 binding to recombinant gp120 IIIB and BaL, respectively, by pomegranate juice (PJ). **Recombinant gp120 coated wells were incubated with dilutions of the PJ for 1 h at 37°C. After removal of the juice, and washing the wells, biotinyl-CD4 was added, and its binding to the wells was measured by ELISA.

Blocking virus entry is a primary target for microbicide development [3,8-11]. Therefore, it was of interest to determine whether or not PJ inhibited the binding of the HIV-1 envelope glycoprotein gp120 to CD4, the common receptor for both X4 and R5 viruses. Pretreatment of both gp120 IIIB and BaL by PJ inhibited subsequent binding of soluble labeled CD4 (Fig. 2). This suggested that one or more PJ ingredients bound strongly or irreversibly to the CD4 binding site on gp120. These results, obtained in an ELISA using gp120 immobilized on polystyrene plates, were confirmed in another assay in which both gp120 and CD4 were in soluble form (data not shown). In reverse experiments, pretreatment of CD4 with PJ failed to block subsequent gp120 binding. Other juices having anti-HIV-1 activity (blueberry, cranberry, grape and lime) failed to block gp120-CD4 binding.

**Figure 3:**
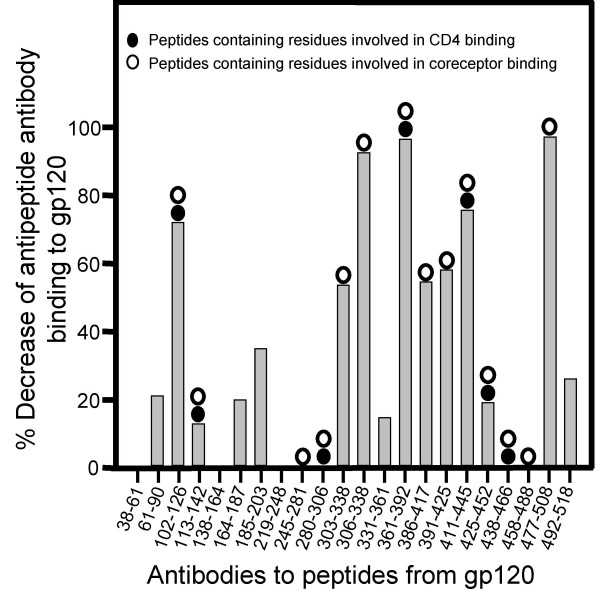
**Inhibition by pomegranate juice (PJ) of binding to gp120 of antibodies to synthetic peptides from the gp120 sequence. **Wells of polystyrene plates coated with gp120 IIIB were incubated with 4-fold diluted PJ for 1 h at 37°C. After removal of PJ, the wells were washed, and 50-fold diluted anti-peptide antisera [21] were added. Bound IgG was quantitated by ELISA. PJ was not added to control wells. Decreases of absorbance, as compared to the respective control wells, are plotted.

**Figure 4:**
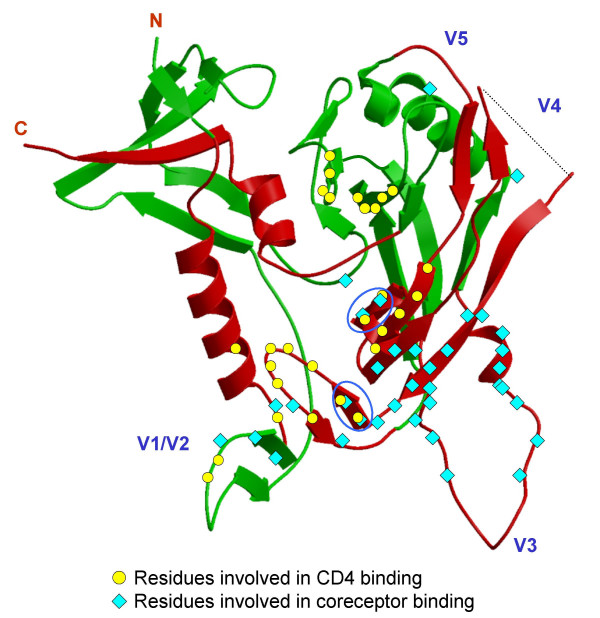
**Location on the gp120 structure of segments corresponding to anti-peptide antibodies whose attachment to gp120 is inhibited by ≥ 50 % in the presence of pomegranate juice (red) and of amino acid residues involved in CD4 and CXCR4/CCR5 coreceptor binding, respectively. **Green, portions of the structure corresponding to anti-peptide antibodies whose attachment to gp120 is not significantly inhibited by PJ. The CD4 domains and the antigen-binding fragment of a neutralizing antibody were excised from the structure of the gp120-CD4-antibody complex [31] (1gc1 retrieved from the Protein Data Bank (pdb) [http://www.rcsb.org/pdb/). The V3 loop, generated by homology modeling, was added to the gp120 structure as described [16]. The figure was generated by Molscript [71] and Raster3D [72,73]. The locations of gp120 variable loops (V1 – V5) and of the N- and C-termini of the sequence are indicated.

To delineate sites on gp120 blocked by the PJ inhibitor(s), the inhibitory effect of PJ on binding to gp120 IIIB of antibodies to peptides derived from the amino acid sequence of gp120 was studied. The binding of antibodies to peptides (102–126), (303–338), (306–338), (361–392), (386–417), (391–425), (411–445) and (477–508) was significantly (≥ 50%) inhibited (Fig. 3). The binding to gp120 IIIB of monoclonal antibodies 9284 and 588D, specific for the gp120 V3 loop (residues 303 – 338) and the CD4 binding site, respectively [29,30] was each inhibited by 97%. Some of the relevant peptides contain residues involved in CD4 binding [31-33] while all discerned peptides include residues involved in coreceptor binding [34-39]. The locations of the peptides and of residues involved in receptor/coreceptor binding on the X-ray crystallographic structure of gp120 are shown in Fig. 4. These results suggest that the PJ inhibitor(s) may also block gp120-coreceptor binding. This will be addressed subsequently.

### Separation of anti-HIV-1 inhibitor(s) from pomegranate juice

**Figure 5:**
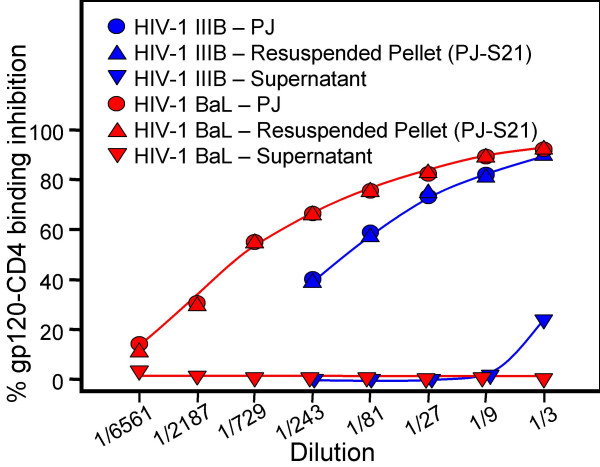
**Adsorption onto corn starch of gp120-CD4 binding inhibitor(s) from pomegranate juice (PJ). **Corn starch (PURITY^® ^21, NF grade; 200 mg/ml) was added to PJ prefiltered to remove particulates. After mixing for 1 h at ≈20°C, the starch was allowed to settle and the supernatant fluid was removed by aspiration. The pellets, resuspended (200 mg/ml) in phosphate buffered saline, and the supernatant fluids were tested at serial dilutions for inhibition of CD4 binding to gp120 IIIB as described in the legend for Fig. 2. The inhibitory activity of the resuspended pellet against gp120 BaL-CD4 binding was then confirmed. Control starch did not inhibit gp120-CD4 binding.

PJ is intensely colored. Therefore, it cannot be directly formulated into a microbicide since it would stain clothing, which is unacceptable. Attempts were made to separate or isolate the active ingredient(s) from PJ. After striving intermittently for over four years to accomplish this, it was discovered that the inhibitor(s) of gp120-CD4 binding can be adsorbed effectively (≥ 99%) onto a selected brand of corn starch (Fig. 5), resulting in a nearly colorless product, designated as PJ-S21. PJ-S21, suspended in water or unbuffered 0.14 M NaCl had a pH of 3.2, compatible with the acidic vaginal environment in which it would remain stabile after application (see below). Inhibitors of gp120-CD4 binding could be eluted from PJ-S21 by extraction with ethanol/acetone 6:4. Drying of the extract followed by gravimetry indicated that the extract contained 3.17 mg solids per gram of PJ-S21.

**Figure 6:**
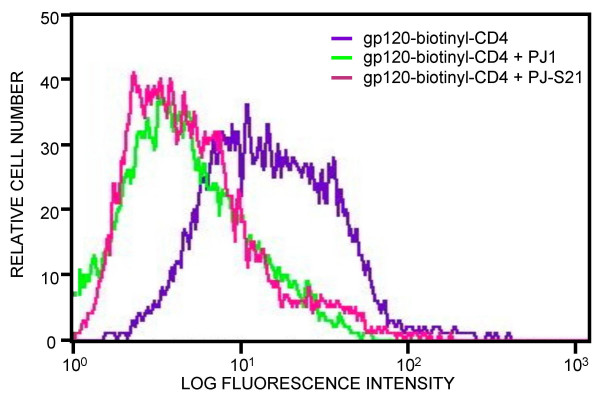
**Inhibition by pomegranate juice (PJ) and PJ-S21, respectively, of gp120 IIIB-CD4 complex binding to cells expressing CXCR4 coreceptors. **HIV-1 IIIB gp120 (5 μg) and biotinyl-CD4 (2.5 μg) were added to 100 μl phosphate buffered saline (PBS) containing 100 μg/ml bovine serum albumin (BSA) (PBS-BSA) and PJ (final 3-fold dilution) or PJ-S21 (67 mg; corresponding to 212 μg solids from PJ adsorbed onto starch). After 1 h at 20°C, the respective mixtures were added to 10^6 ^MT-2 cells. After 30 min, the cells were washed 3 times with PBS-BSA and PE-streptavidin (a fluorescent label specific for biotin; 0.1 μg) was added. After 20 min, the cells were washed and fixed by 1% formaldehyde in PBS. Flow cytometry analysis was performed in a FACSCalibur flow cytometer (Becton Dickinson Immunocytometric Systems, San Jose, CA). The median relative fluorescence values for cells exposed to gp120-CD4; gp120-CD4 + PJ; gp120-CD4 + PJ-S21; and control cells were: 13.7; 4.0; 4.3; and 2.1, respectively.

**Figure 7:**
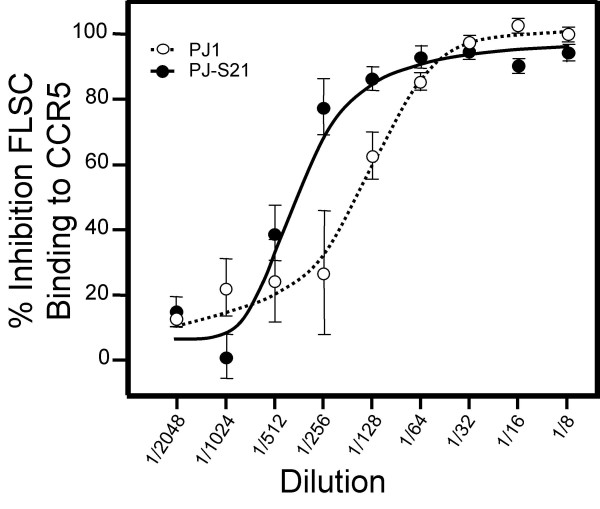
**Inhibition by pomegranate juice (PJ) and PJ-S21, respectively of FLSC binding to CCR5 expressing Cf2Th/synCCR5 cells. **FLSC is a chimeric recombinant protein consisting of gp120 BaL linked with D1D2 domains of CD4. The inhibitory effect was quantitated using a cell-based ELISA [20]. The starting concentration of PJ-S21 was 200 mg/ml, corresponding to 634 μg/ml solids adsorbed onto starch from PJ.

**Figure 8:**
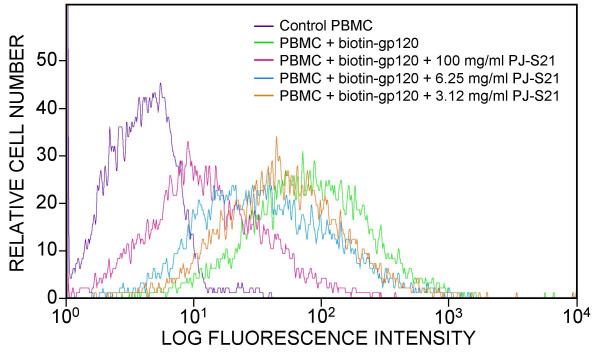
**Inhibition by PJ-S21 of biotinyl-gp120 IIIB binding to peripheral blood mononuclear cells (PBMCs). **HIV-1 IIIB biotinyl-gp120 (5 μg) was added to 100 μl of PBS-BSA containing graded quantities of PJ-S21. After 1 h at 20°C, the respective mixtures were added to 10^6 ^PBMCs. After 30 min the cells were washed 3× with PBS-BSA and PE-streptavidin (0.1 μg was added). Subsequently the procedures described in the legend to Fig. 6 were used. The median relative fluorescence values for control cells and cells exposed to biotinyl-gp120 in the absence and presence of PJ-S21 (100, 6.25 and 3.12 mg/ml) were 4.1, 81.31, 12.2, 35.2 and 50.0 respectively. 100 mg of PJ-S21 corresponds to ≈ 320 μg solids adsorbed from PJ onto starch.

**Figure 9:**
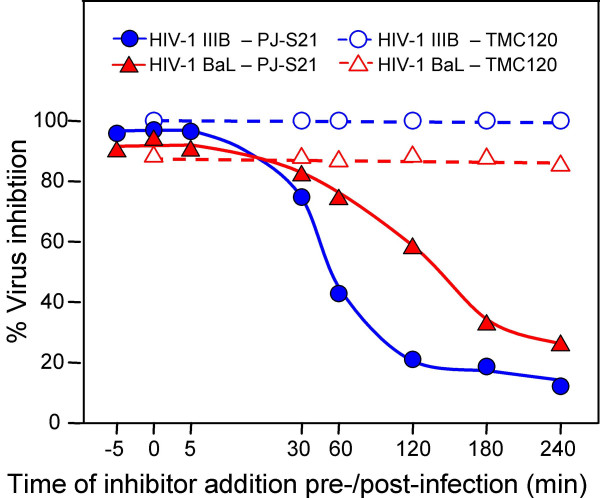
**Inhibition of HIV-1 IIIB or BaL replication depends on the time of PJ-S21 addition pre- or post-infection. **For comparison, the inhibition of infection by the nonnucleoside reverse transcriptase inhibitor TMC-120, added to cells at distinct intervals after HIV-1, was determined (dotted lines). Virus infection was measured by quantitation of β-galactosidase.

PJ-S21, to the same extent as the original PJ, inhibited the binding of gp120 IIIB-CD4 complexes to cells expressing CXCR4, as determined by flow cytometry (Fig. 6). Similarly, binding of a gp120 BaL-CD4 fusion protein to cells expressing CCR5 was blocked by PJ and PJ-S21, as detemined by a cell based ELISA [20]; (Fig. 7). Thus, PJ-S21 is an inhibitor of both X4 and R5 virus binding to the cellular receptor CD4 and coreceptors CXCR4/CCR5. PJ-S21 also inhibited gp120 binding to PBMCs as determined by flow cytometry (Fig. 8). To confirm that PJ-S21 functions as a virus entry inhibitor, the complex was added to cells at time intervals before and after infection of cells by HIV-1 IIIB and BaL, respectively. Results shown in Fig. 9 demonstrate that PJ-S21 interferes with early steps of the virus replicative cycle.

To be considered as a topical microbicide, PJ-S21 must be formulated to withstand storage in a tropical environment. Accelerated thermal stability studies revealed that a water suspension of PJ-S21 maintained only 4, 11, and 33%, respectively, of its original activity (measured by inhibition of gp120-CD4 binding) when stored for 30 min at 60°C, and one week at 50°C or 40°C. On the other hand, a dried PJ-S21 powder remained fully active after storage at 50°C for 12 weeks (the longest time used in the evaluation). Consequently, anhydrous formulations should be preferred for further development.

**Figure 10:**
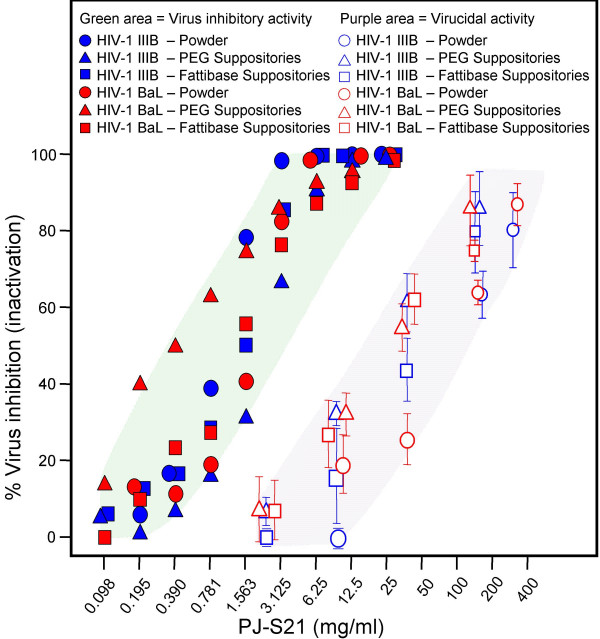
**HIV-1 inhibitory and virucidal activity of PJ-S21 and its formulations. **Inhibition of infection by HIV-1 IIIB and BaL, respectively, was determined as described in the legend for Fig. 1. To measure virucidal activity, the respective viruses were mixed with graded quantities of PJ-S21 for 5 min at 37°C. After low speed centrifugation, the viruses were separated by precipitation with PEG 8000 and centrifugation. The resuspended pellets and control untreated viruses were serially diluted, and the dilutions assayed for infectivity. The concentration range given on the abscissa corresponds to 0.31 – 1,268 μg solids adsorbed from PJ to starch.

Three such formulations were prepared: two kinds of suppositories, melting at 37°C, and a tablet (for compositions see Methods section). The inhibitory activity of PJ-S21 was fully preserved after 12 weeks storage at 50°C within tablets, and at 30°C within the suppositories (the highest temperature considered to prevent melting). Data showing the inhibition of infection by HIV-1 IIIB and BaL respectively, by PJ-S21 and its formulations (except the tablets which also contain anti-HIV-1 inhibitors other than PJ-S21, i.e. Carbopol 974P [18]) are summarized in Fig. 10. Their inhibitory activities against HIV-1 IIIB and BaL were similar, unlike the inhibitory activities of the original PJs (Fig. 1). These formulations were also virucidal, albeit at concentrations higher than those sufficient for inhibition of infection. These experiments also revealed that PJ-S21 was not cytotoxic under the experimental conditions used. The inhibitory/virucidal activities were maintained in the presence of seminal fluid (SF) at a 1:1 (w/w) ratio of SF to PJ-S21; (data not shown).

A microbicide can be considered potentially successful, only if it has antiviral activity against primary virus isolates belonging to distinct virus clades and phenotypes. PJ-S21 meets this requirement since it inhibited infection by primary HIV-1 strains of all clades tested having R5 and X4R5 (= dual-tropic) phenotypes (Table 1).

## Discussion

**Figure 11:**
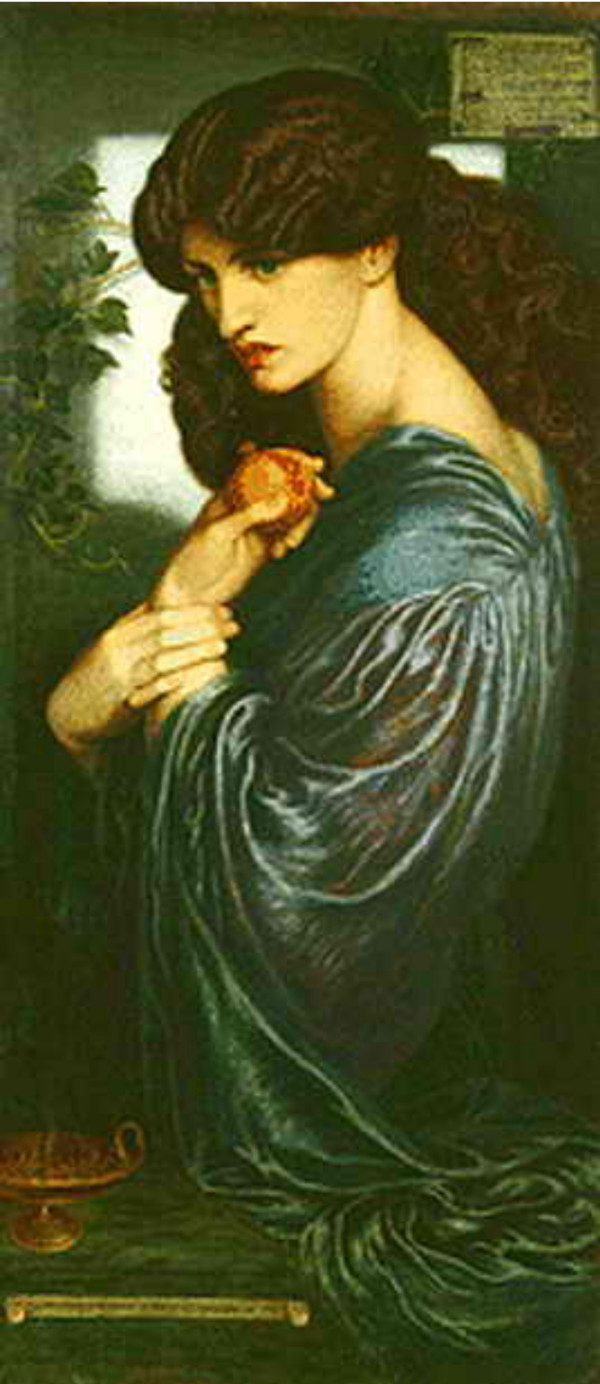
**Persephone (IIερσεφονη) holding a pomegranate. **In Greek mythology she had eaten seeds of the fruit and consequently was condemned to remain in Hades, the Underworld, for six months of every year. Derived from a painting of Dante Gabriel Rossetti in the Collection of the Tate Gallery in London.

Pomegranates have been venerated for millennia for their medicinal properties and considered sacred by many of the world's major religions. In deference to pomegranates, the British Medical Association and several British Royal Colleges feature the pomegranate in their coat of arms. The Royal College of Physicians of London adopted the pomegranate in their coat of arms by the middle of the 16^th ^Century [40]. The best known literary reference to the contraceptive power of pomegranate seeds is classical Greek mythology. Persephone (IIερσεφονη) had eaten six pomegranate kernels (from which juice is derived) while in the Underworld and for that many months the land remained infertile during the Fall and Winter (Fig. 11). Ironically, this report shows that pomegranate juice contains HIV-1 entry inhibitors targeted to the virus envelope corresponding to a class of anti-retroviral drugs still scarce in development [41].

PJ contains several ingredients [42,43] which, isolated from natural products other than PJ, were reported to have anti-HIV activity, for example: caffeic acid [44], ursolic acid [45], catechin and quercetin [46,47]. However, these compounds, in purified form, obtained commercially, did not block (at 200 μg/ml) gp120-CD4 binding as measured by the ELISA described above and did not adsorb to corn starch, unlike the entry inhibitor(s) from PJ. In fact, the supernatant after treatment of PJ with starch, and removal of the entry inhibitors, retained anti-HIV-1 activity and also inhibited infection by herpes virus type 1, unlike the HIV-1 entry inhibitors which adsorbed onto starch. Thus, the antiviral activities in the supernatant appeared to be non-specific, and probably similar to those of extracts from pomegranate rind [48,49], and were not characterized further. Additional information [50-53] has revealed that the findings apply to crude extracts from pomegranate rind prepared at elevated temperatures under conditions which destroy the HIV-1 entry inhibitor described here.

The inhibitor(s) interfering with gp120 binding to CD4 (Fig. 2 and 5) blocked additional sites on gp120 (Fig. 3) involved in interaction with the CXCR4/CCR5 coreceptors (Fig. 4, 6 and 7). This was not completely expected and can be explained either by the presence of multiple inhibitors with distinct or overlapping specificities in PJ-S21 or by induction of gp120 conformational changes [54] resulting in blockade of both CD4 and CXCR4/CCR5 binding sites on gp120. Similar effects have been noticed for other small molecule inhibitors [55]. Simultaneous blocking of more than a single site on HIV-1 involved in virus entry is expected to increase the effectiveness of candidate microbicides [11]. The target sites for the inhibitor(s) are likely to be located within the protein moiety of gp120 since binding of labeled *Galanthus nivalis *lectin (specific for terminal mannose residues [56]; and other lectins to gp120 oligosaccharides was not diminished in the presence of PJ or PJ-S21 (data not shown).

Blocking of CD4 binding sites on HIV-1 gp120 by monoclonal antibodies or a CD4-IgG2 recombinant protein has been shown to be sufficient to inhibit HIV-1 infection of human cervical tissue *ex vivo *[11] and in preventing virus transmission to macaque monkeys when applied vaginally [57]. Therefore, it seems likely that PJ-S21 will be similarly effective, an expectation which remains to be confirmed.

The application of PJ-S21 as a topical anti-HIV-1 microbicide requires reasonable uniformity among batches produced at distinct times and locations. Similarities in gp120-CD4 binding inhibitory activity among distinct freshly prepared and commercial juices stored for unknown periods (Fig. 2) suggest that this should be feasible. Pasteurization of juice for 30 seconds at 85°C resulted in complete loss of inhibitory activity. A commercial PJ concentrate exposed to 61°C, and two other concentrates, presumably prepared by evaporation at elevated temperatures, had no or drastically diminished activity. The gp120-CD4 inhibitory activity from PJ3 (juice with fructose and citric acid added), failed to bind to starch. Separate experiments revealed that these compounds interfere with inhibitor binding to corn starch. Therefore, PJs intended for production of the PJ-S21 complex must be sterilized by filtration and be free of additives.

Particular attention must be devoted to the selection of starch, a pharmaceutical excipient generally used in vaginal formulations [58], for effective binding of the virus entry inhibitors from PJ. Among a dozen starches tested, the best results have been obtained with S21. With other brands, the adsorption of the inhibitors was either incomplete or their binding did not result in a complex having activity in the ELISA measuring gp120-CD4 binding inhibition (ARGO^® ^corn starch), presumably, because of irreversible binding of the PJ inhibitors. Interestingly, there are only a few references available regarding the use of starch as an adsorbent for different compounds: flavors [59,60], dyes [61-63], low-molecular mass saccharides [64], lipids [65,66], proteins [67] and iodine [68].

The intended dose of PJ-S21 for vaginal application is 1.0 to 1.5 g, (= 3.17 – 4.76 mg solids from PJ adsorbed onto starch) i.e. ≥ 100-fold higher than the dose needed for blocking HIV-1 infection *in vitro *(Fig. 10, Table 1), and thus expected to meet requirements for likely *in vivo *protection against vaginal challenge [69]. This quantity of PJ-S21 is produced from 5 to 7.5 ml of PJ, i.e. ≤ 5% of a single (150 ml) serving of juice, attesting to the safety, feasibility and economy of this proposed candidate topical microbicide.

In an alternative approach to formulation development, PJ-S21 can be incorporated into a water dispersible film (similar to the widely available "breath control" strips) or into water dispersible sponges [70] which are converted into a gel following topical application [19]. Each of the above formulations would meet the following requirements: (1) minimization of waste disposal problems associated with the use of applicators needed for delivery of microbicidal gels/creams; (2) simplicity; (3) small packaging and discretion related to purchase, portability and storage; (4) low production costs; (5) amenability to industrial mass production at multiple sites globally and (6) potential application as rectal microbicides. Furthermore, it would remain possible to produce for local use PJ-S21 based gel formulations with a limited shelf life, avoiding the costs of producing dry PJ-S21 powders via appropriate low temperature drying processes. Whichever of these formulations is selected, adequate quality control will be needed to assure uniform anti-HIV-1 activity of the final product(s) and to establish reproducible conditions for manufacture.

## Conclusions

PJ-S21 can be classified as an AAAA candidate microbicide: Acceptable; Accessible; Affordable; and Accelerative in transition from development to marketing. Thus, PJ-S21 would be expected to circumvent some problems associated with antiretroviral drugs and possibly some of the other candidate microbicides, i.e. uncertainty related to potential side effects, investment and time needed to establish inexpensive large scale production, and monopoly of supply.

## Abbreviations used

AIDS, acquired immunodeficiency syndrome; BSA, bovine serum albumin; ED_50(90)_, effective dose(s) for 50% (90%) inhibition of infection; ELISA, enzyme linked immunosorbent assays; FBS, fetal bovine serum; FLSC, a full length single chain protein consisting of BaL gp120 linked with the D1D2 domains of CD4 by a 20 amino acid linker; HIV-1, human immunodeficiency virus type 1; HRP, horseradish peroxidase; LTR, long terminal repeat; PBMCs, peripheral blood mononuclear cells; PBS, phosphate buffered saline; PEG, polyethylene glycols; PJ, pomegranate juice; S21, PURITY^® ^21 corn starch NF grade; SF, seminal fluid.

## Competing interests

The authors declare that they have no competing interests.

## Authors' contributions

ARN developed the concepts representing the basis of the manuscript and designed most experiments. NS contributed to the development of experimental techniques and carried out experiments other than infectivity assays. YYL did all the tissue culture work and viral infectivity assays. AKD did all the molecular modeling studies and contributed to the development of cell based enzyme linked immunosorbent assays.

## Pre-publication history

The pre-publication history for this paper can be accessed here:



## Figures and Tables

**Table 1:** Inhibitory activity of PJ-S21 on infection by primary HIV-1 strains

Primary strain	Subtype, Coreceptor use	ED_50_* mg/ml	ED_90_* mg/ml
92RW008	A, R5	0.50 ± 0.05	2.76 ± 0.28
94UG103	A, X4R5	1.42 ± 0.54	3.42 ± 0.98
92US657	B, R5	0.62 ± 0.11	2.86 ± 0.33
93IN101	C, R5	3.56 ± 1.10	8.87 ± 2.55
93MW959	C, R5	1.02 ± 0.19	3.54 ± 0.90
92UG001	D, X4R5	0.62 ± 0.17	2.94 ± 0.85
93THA051	E, X4R5	0.86 ± 0.01	4.09 ± 0.08
93BR020	F, X4R5	4.25 ± 0.78	8.31 ± 1.04
RU570	G, R5	0.42 ± 0.09	1.54 ± 0.16
BCF02	Group O, R5	0.59 ± 0.29	3.92 ± 0.27

* ED_50(90) _= effective dose(s) of PJ-S21 for 50% (90%) inhibition of infection. One gram of PJ-S21 contains approximately 3.2 mg of the inhibitors adsorbed to starch from pomegranate juice.
